# Relationship between Body Mass Index, Skeletal Maturation and Dental Development in 6- to 15- Year Old Orthodontic Patients in a Sample of Iranian Population

**Published:** 2014-12

**Authors:** Zohreh Hedayati, Fatemeh Khalafinejad

**Affiliations:** a Orthodontic Research Center, Dept. of Orthodontic, School of Dentistry, Shiraz University of Medical Sciences, Shiraz, Iran.; b Post graduate Student, Orthodontic Research Center, School of Dentistry, Shiraz University of Medical Sciences, Shiraz, Iran.

**Keywords:** Body mass index, Skeletal maturation, Dental development, Cervical vertebral method, Dental age

## Abstract

**Statement of the Problem:** The prevalence of overweight and obesity has been increasing markedly in recent years. It may influence growth in pre pubertal children.

**Purpose:** The purpose of this study was to determine whether increased Body Mass Index (BMI) is associated with accelerated skeletal maturation and dental maturation in six to fifteen years old orthodontic patients in Shiraz, Iran.

**Materials and Method:** Skeletal maturation and dental development of 95 orthodontic patients (65 females and 30 males), aged 6 to 15 years, were determined. Dental development was assessed using the Demerjian method and skeletal maturation was evaluated by cervical vertebral method as presented by Bacetti. The BMI was determined for each patient. T-test was applied to compare the mean difference between chronologic and dental age among the study groups. A regression model was used to assess the relationship between BMI percentile, skeletal maturation, and dental development.

**Results:** 18.9% of subjects were overweight and obese. The mean differences between dental age and chronologic age were 0.73±1.3 for underweight and normal weight children and 1.8±1.08 for overweight and obese children. These results highlighted the correlation between accelerated dental maturity and increasing BMI percentile (*p*= 0.002). A new formula was introduced for this relationship. There was not any significant relationship between BMI percentile and skeletal maturation.

**Conclusion:** Children who were overweight or obese had accelerated dental development whereas they did not have accelerated skeletal maturation significantly after being adjusted for age and gender.

## Introduction

The prevalence of overweight and obesity in most developed and developing countries has been markedly increasing in recent years. This is seen among all genders, ages, racial, ethnic groups, and educational levels.[1] Some important risk factors in obesity are urbanization, reduced physical activity, increased energy intake, and the modern life style.[2] In Iran, the prevalence of overweighting in children aged 6 to 18 years has been doubled from 4.2 percent to 8.3 percent between 1993 and 1999.[3] The prevalence of obesity in Iran (1997 to 2007) has been 5.5% in children (<18years) and 21.5% in adults.[2]

Body mass index (BMI) is the preferred method of expressing body fat percentile in epidemiologic studies.[1] BMI is calculated by dividing weight by squared height: BMI= mass (kg) / (height (m))^2^.[4]

BMI calculation is different for individuals that are under twenty years old. It is calculated the same as adults, but it is compared with typical values for other children of the same gender and age (BMI percentile), allowing comparison with children of the same age and gender.

A BMI less than 5^th^ percentile is considered as underweight and above the 95^th^ percentile as obese. A BMI between 85^th^ and 95^th^ percentile is considered to be overweight and a BMI between 5^th^ and 85^th^ is deemed as a normal weight.[4]

Childhood obesity can be related to hypertension, type II diabetes, dyslipidemia, left ventricular hypertrophy, obstructive sleep apnea, orthopedic, and psychological problems.[5] An earlier onset of puberty has been reported in children with higher BMI percentile.[6-7] Sadeghianrizi et al. reported that obese individuals may have increased maxillary and mandibular length (maxillary and mandibular prognathism) compared with the control group.[8]

Determination of skeletal maturation and dental development is important in the initial phase of orthodontic treatment, especially in growth modification treatments. To the best of our knowledge; no study investigated simultaneously the relationship between skeletal maturity and the dental development with increased obesity in Iranian children. Our study was conducted aiming to assess the relationship between BMI, skeletal maturation, and dental development in 6-15 years old orthodontic patients in Shiraz. 

## Materials and Method

The protocol for this cross-sectional study was approved by the Orthodontic Research Center of Shiraz Dental School. Ethical approval was taken from ethical committee of Shiraz University of Medical Sciences (#CT-p-92-9989).

Our study sample consisted of 95 patients (65 females and 30 males), aged between 6-15 years old, all selected from referred patients to Shiraz orthodontic clinics from 2012.9.23 to 2013.3.14. In the first visit, an informed consent was obtained from patients (or their parents). The patients’ records consisted of panoramic and lateral cephalometric radiographs. The anthropometric measurements were taken by one of the investigators at the first appointment. The patients’ weights (kg) were measured to the nearest 0.1 kg using a mechanical scale and their heights (cm) using a wall-mounted stadiometer (Invernizzi; Rome, Italy) to the nearest 1 cm. The inclusion criteria comprised of:

1) Adequate diagnostic quality of panoramic and lateral cephalometric radiographs.
2) Presence of all permanent mandibular teeth except for the third molars.
3) Visibility of the second, 3^rd^ and 4^th ^cervical vertebrae in patients’ lateral cephalometric radiographs.
4) Ages greater than or equal to 6 years and less than or equal to 15 years.

Evaluated by two expert dental radiologists, the patients with any congenital tooth anomalies or congenital anomalies of the 2nd, 3rd and 4^th^cervical vertebrae such as fusion between cervical vertebrae or presence of secondary ossicle were eliminated. Patients having any systemic diseases that could affect growth (such as nutritional disturbance, endocrine disorders, syndromes, and long term consumption of medication) were excluded from the investigation.

BMI was calculated as weight divided by height squared: BMI= mass (kg)/(height(m))^ 2^ .The BMI score, age, and gender were used to obtain the BMI percentile value for each subject with age- and gender-specific growth charts from the Centers for Disease Control (CDC).[9-10]

It has been reported that the cut-off points used by the US Centers for Disease Control and Prevention (CDC) were more feasible for Iranian children compared to the other definitions.[11-12]

The dental age was determined using the method codified by Demerjian who used a single panoramic radiograph to evaluate the seven teeth of the left mandibular segment. The development of the roots and crowns of these teeth were categorized into different groups, ranging from a (least development) to H (complete development) where each letter grade was associated with an experimentally determined maturity score based on the specific tooth and the gender of the patient. The maturity scores were then summed up to produce an overall maturity score correlated with a specific dental age.[13]

The skeletal age was determined by using cervical vertebra method (CVM), assessing the cervical vertebrae shape and inferior border concavity of c_2_-c_4 _presented by Bacetti et al. in 2005.[14] The six cervical vertebra development stages were then divided into two groups: 1 to 3 into pre-spurt and 4 to 6 into post-spurt.[15]

Panoramic and lateral cephalometric images were evaluated on a view box in a dark room. Twenty panoramic and lateral cephalometric radiographs were selected randomly and re-evaluated two weeks later.

**Statistical analysis**

The analyses were performed using the SPSS software (version 16; SPSS Inc., Chicago IL, USA). Intra examiner reliability was determined by using Cronbach’s alpha test.

Independent sample t-test was performed to compare the mean chronologic age, dental age, and BMI percentile among boys and girls. We applied Chi-squared test to compare the mean skeletal age in girls and boys. To assess the correlation between chronologic age and dental age by gender, we employed Pearson’s correlation test. The Spearman's correlation test was used to determine the correlation between skeletal age and chronologic age and the correlation between skeletal maturity and dental age. The mean difference comparison between chronologic age and dental age among boys and girls was conducted by using paired sample t-test. The subjects were then classified into two groups (one group underweight and normal and the other group overweight and obese) and t-test was employed to compare the mean difference between chronologic age and dental age between the two groups. Multiple linear regression tests were used to assess the relationship between age, gender and BMI percentile with dental age. The logistic regression test was used to assess the relation of age, gender and BMI percentile with skeletal age. 

## Results

From all the patients examined, 95 patients met all the inclusion criteria and entered our study. Approximately 68.4% of samples were female and 31.6% male which simply means that more than half of the samples were girls. The mean chronologic age of the participants was 11.35±1.94 years and the mean dental age obtained was 12.28±2.48 years.

The intra-observer reliability showed a high degree of consistency between the two evaluations. Cronbach^’^s alpha was 0.94 for measuring the skeletal ages and 0.96 for measuring the dental ages for the twenty patients which were randomly selected.

**Figure 1:**
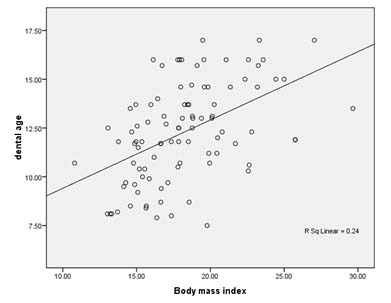
The regression analysis of BMI and dental age in our study sample

The prevalence of overweight and obese patients was 18.9% and that of the underweight was only 12.6 % (Figure 1).

There were no statistically significant differences in mean values between boys and girls in their chronologic age, dental age, and BMI percentile (*p*= 0.422, *p*= 0.693 and *p*= 0.455 respectively) (Table 1).

**Table 1:** The mean chronologic age, dental age and BMI percentile among boys and girls

	**Male (n=30) Mean (±SD)**	**Female (n=65) Mean (±SD)**	**P value**
Chronologic age	11.59 (±1.64)	11.24 (±2.07)	0.422
Dental age	12.43 (±2.6)	12.21(±2.44)	0.693
BMI percentile	45.27 (±38.23)	50.71(±30.09)	0.455

As the results of the study indicated, while the most of boy subjects were in pre-spurt stage (stages 1, 2, 3), the most of the girls were placed in post- spurt stage (stages 4, 5, 6). None of the boys were in stage 6. However, girls were more advanced in skeletal age than boys (*p*= 0.004) (See Table 2). It was noticed that there was a direct relationship between chronologic age and dental age (r= 0.841, *p*≤ 0.001), chronologic age and skeletal age (r= 0.717, *p*≤ 0.001), and also dental age and skeletal age (r= 0.658, *p*< 0.001).

**Table 2:** Sex in relation to CVM stage

**Sex**	**CVM category Pre-spurt (stages 1-3)**	**Post-spurt (stages 4-6)**	**P value**
Male	76.7%	23.3%	
Female	43.1%	56.9%	0.004
Total	53.7%	46.3%	

The mean difference between chronologic age and dental age was higher in girls (0.97±1.24 y) than in boys (0.84±1.57 y) (Table 3). The mean difference between chronologic age and dental age was significantly higher in overweight and obese patients than underweight and normal weight patients (*p*= 0.002) (See Table 3).

**Table 3:** Comparison of mean difference between dental age and chronologic age by sex and BMI percentile

**Weight**	**Mean (±SD)**	**P value**
Underweight and normal weight	0.72 (±1.32)	0.002
Overweight and Obese	1.82 (±1.08)	0.002
Male	0.84 (±1.57)	0.005
Female	0.97 (±1.24)	< 0.001
Total	0.93 (±1.35)	< 0.001

Regression analysis revealed that chronologic age and BMI percentile were the statistically significant explanatory variables for the dental age (*p*< 0.001) (Figure 1). In the initial model for dental age, gender did not contribute significantly to the age and BMI percentile (Table 4).

**Table 4:** Regression model results for dental age

**Parameters**	**Estimate**	**Beta**	**T**	**P value**
Constant	-0.8	-	-0.862	0.391
Age	1.06	0.838	16.24	< 0.001
BMI Percentile	0.017	0.228	4.48	< 0.001
Sex	0.059	0.011	0.214	0.841

BMI percentile was not a statistically significant explanatory factor for cervical vertebral maturation. Gender and age had the largest estimated parameter value in this regard (Table 5).

**Table 5:** Logistic model results for cervical vertebral maturation

**Parameter**	**Estimate**	**OR**	**95% for OR**	**P value**
Sex	3.73	42.04	5.75-306.93	<0.001
Age	1.62	5.091	2.45-10.55	<0.001
BMI percentile	0.012	1.012	0.99-1.03	0.231
Constant	-22			<0.001

## Discussion

Pediatric obesity is growing in many countries throughout the world, including Iran. In a meta-analysis study analyzing 58 articles between years 1997 to 2007, the prevalence of obesity among Iranian children under 18years of age was 5.5% and its prevalence was slightly greater in boys than girls.[2] The prevalence of obesity was 6.3% showing higher values in recent years in Iran. Boys had higher prevalence of overweight than girls (16.7 versus 10.8), which was not statistically significant (*p*= 0.097).

Dental age was determined using the method codified by Demerjian. The mean difference between dental age and chronologic age (0.93+1.35 years) was statistically significant (*p*< 0.001). Many studies such as those conducted in Iran,[16] England,[17] Turkey,[18] Saudi Arabia,[19] Poland,[20] Newzealand[21] and Korea[22] showed that the Demerjian method would overestimate the age. Demerjian^,^s system of dental age assessment was carried out on 2928 patients of French Canadian parentage in 1973.[13] These findings however, may be different in other countries because of different race, diet and socioeconomic conditions.

In our study, girls had more advanced dental age. These results reveal more consistency with the findings of Hilgers et al. who found that dental development acceleration was significantly greater for girls than boys (boys were accelerated 1.21 years and girls 1.52 years).[23] Teivens et al. also, reported similar results.[24] Mack et al. found a greater average advancement in boys than in girls.[15] This could be the effect of the extrapolation to adjust the dental maturity scores for boys that had dental ages greater than 16.

In our study, there was a direct relationship between dental age and chronologic age (r= 0.841, *p*≤ 0.001). Chaudhry et al. reported a strong correlation between chronologic age and dental age (r= 0.650) in Indian girls.[25] Zangoui-Booshehri et al. reported the similar high relationship between chronological and dental age (r =0.784).[26]

The mean differences between dental age and chronologic age were 0.73±1.3 for underweight and normal weight children and 1.8±1.08 for overweight and obese children. These results highlighted the correlation between accelerated dental maturity and increasing BMI percentile as observed in other studies.[16, 23] In contrast, Eid et al. did not find a significant correlation between dental maturity and body mass index in Brazilian children[27] that could be attributed to genetic and/or dietary factors.

In the initial model for dental age, sex did not contribute significantly to the age and BMI percentile. It could have been due to the lower number of boys than girls in our study. The expected change in dental age for a single unit change in BMI percentile was 0.017. The following is an achieved formula to calculate dental age presented in Table 4.

Dental age = 1.06 chronologic age + 0.017 BMI percentile

For instance, a 10 year old girl with a BMI percentile of 85 would be estimated to have dental age of 12. Therefore, it can be estimated that for a patient with 95^th^ BMI percentile, we should begin growth related orthodontic treatment 6 months earlier than that for a patient with a 65^th^ BMI percentile of the same gender and age.

In our study, the mean chronologic age was not significantly different among boys and girls but girls demonstrated a statistically significant increase in the prevalence of advanced cervical vertebral maturation stage. Results of this study showed that girls were more avant-garde in skeletal maturation than boys. Akridge et al. assessed skeletal maturation with Fishman’s hand-wrist analysis. Their results also showed that skeletal maturation was more accelerated among girls than boys.[28] Basaran et al. demonstrated that girls compared with boys reached the cervical vertebral maturation stages 1 through 5 at younger ages. They, however, reached stage 6 at older ages than boys.[29]

We found a direct relationship between chronologic age and skeletal age (r= 0.717, *p*≤ 0.01).These findings were consistent with those of Bacetti et al. and Mack et al.[14-15] but Alkhal et al. reported a low correlation between chronologic age and cervical vertebral maturation in southern Chinese children.[30] The CVM method introduced by Bacetti et al. examined 706 patients in Michigan university in 2005.[14] The different results could have been due to ethnic differences.

We did not find a significant relationship between BMI percentile and cervical vertebral maturation. Gender and age were the most important parameters for estimating the skeletal age (Table 5). Flores-Mir et al. using Fishman^'^s analysis, evaluated the association between growth stunting and skeletal maturation in Peru. Like our study they did not find a significant difference for the skeletal maturation according to nutritional status.[31] Also Akridge et al. found that acceleration in skeletal age did not significantly increase with the rise in the BMI.[28] However, there are many studies that report a significant increase in skeletal age by increasing BMI percentile.[15, 32-34] It seems a larger sample size with greater number of obese children is needed to demonstrate this relationship confidently.

Despite the results achieved, the current study is suffered from some limitations. We used Demerjian method to calculate dental age, but this method was introduced by Demerjian several years ago for French Canadian children. Additionally, we used the cervical vertebral method for assessing skeletal age. Bacetti presented the CVM atlas based on lateral cephalometric radiographs of American children whom are different from Iranian children in terms of race, diet, and socioeconomic condition. This study was also limited with its small sample size when separated by gender and BMI status.

Orthodontic treatments usually last 1-2 years or more, therefore, an orthodontist has the opportunity to impact general health status of children and adolescents. However, it is not a routine procedure in Iranian dental clinics and universities to measure the weight and height of the patients. Childhood obesity is related to many different diseases and malfunctions (hypertension, type II diabetes, dyslipidemia, left ventricular hypertrophy, obstructive sleep apnea and orthopedic and psychological problems) [5] and it is the orthodontist^'^s responsibility to orient patients about their health status and its risk factors and refer these obese patients for medical consultation when necessary. Considering orthodontists as a part of the health care providers, they should provide suitable education to improve their patients’ oral health and also body fat status.

Additionally, based on the results of our study, increasing BMI percentile accelerates dental age and may affect treatment timing such as that in serial extraction or space maintenance. When permanent teeth erupt earlier in obese children at a time when they may not have good oral hygiene, the incidence of caries may increase.

Future studies with larger sample sizes are warranted to determine if different chronologic age groups, ethnicity, and socioeconomic condition in coordination with obesity would have an effect on dental and skeletal development. 

## Conclusion

In this sample of orthodontic patients, dental development was accelerated in children who had increased BMI percentile. Overweight or obese children did not demonstrate significantly accelerated skeletal maturation after adjusting for age and gender.

The prevalence of overweight and obesity have increased markedly which might provide the orthodontist an opportunity to measure weight and height as a diagnostic record for orthodontic treatment planning. Considering an orthodontist as a health care provider, such information can be useful to counsel or refer obese patients if necessary. 
